# Overexpression of EZH2/NSD2 Histone Methyltransferase Axis Predicts Poor Prognosis and Accelerates Tumor Progression in Triple-Negative Breast Cancer

**DOI:** 10.3389/fonc.2020.600514

**Published:** 2021-02-16

**Authors:** Bo Gao, Xiumin Liu, Zhengjin Li, Lixian Zhao, Yun Pan

**Affiliations:** Department of Pathology, First Affiliated Hospital of Dali University, Dali, China

**Keywords:** triple-negative breast cancer, EZH2, NSD2, prognosis, progression

## Abstract

Two histone methyltransferases, enhancer of zeste homolog 2 (EZH2) and nuclear SET domain-containing 2 (NSD2), are aberrantly expressed in several types of human cancers. However, the regulatory relationship between EZH2 and NSD2 and their prognostic values in breast cancer (BC) have not been fully elucidated. In this study, we demonstrated that EZH2 and NSD2 were overexpressed in BC compared with benign lesions and normal tissues using tissue microarray, immunohistochemistry, and bioinformatic databases. Both EZH2 and NSD2 expression were associated with pathological grade of tumor and lymph node metastasis. A comprehensive survival analysis using Kaplan-Meier Plotter database indicated that EZH2 expression was negatively correlated with relapse-free survival (RFS), overall survival (OS), distant metastasis-free survival (DMFS), and postprogression survival (PPS) in 3951 BC patients, and NSD2 expression was negatively correlated with RFS and DMFS. Notably, EZH2 and NSD2 expression were coordinately higher in triple-negative breast cancer (TNBC) than that in other subtypes. Stable knockdown of EZH2 using lentiviral shRNA vector significantly reduced the proliferation, migration and invasion abilities of TNBC cell line MDA-MB-231 and MDA-MB-468, and downregulated NSD2 expression as well as the levels of H3K27me3 and H3K36me2, two histone methylation markers catalyzed by EZH2 and NSD2, respectively. By contrast, overexpression of EZH2 using adenovirus vector displayed an inverse phenotype. Furthermore, knockdown of NSD2 in EZH2-overexpressing cells could dramatically attenuate EZH2-mediated oncogenic effects. Bioinformatic analysis further revealed the function and pathway enrichments of co-expressed genes and interactive genes of EZH2/NSD2 axis, suggesting that EZH2/NSD2 axis was associated with cell division, mitotic nuclear division and transition of mitotic cell cycle in TNBC. Taken together, EZH2/NSD2 axis may act as a predictive marker for poor prognosis and accelerate the progression of TNBC.

## Introduction

Breast cancer (BC) is the most common malignancy among females and the incidence of BC has increased steadily in recent decades (1). About 12.2% of all newly diagnosed BC cases and 9.6% of all BC-related death cases worldwide are from China (1). The survival and prognosis of BC patients have been improved recently by surgery, radiotherapy, chemotherapy, endocrinotherapy, and molecular targeted therapy. However, metastasis and recurrence remain the clinical challenges and the leading causes of high mortality rates for BC patients, especially for those with triple-negative breast cancer (TNBC), since TNBC is insensitive to most available therapies (2). Currently, the molecular pathogenesis of TNBC is poorly understood and novel therapeutic targets for TNBC need to be explored. Hence, it is essential to identify biomarkers and therapeutic targets involved in unfavorable progression of TNBC.

Enhancer of zeste homolog 2 (EZH2) is a SET-domain containing histone methyltransferase and the catalytic subunit of the polycomb repressive complex 2 (PRC2) which inhibits gene transcription through trimethylation of Lysine 27 on histone H3 (H3K27me3). Previous studies have demonstrated the dual nature of EZH2, as it can function as a gene activator or repressor involving in cell proliferation, differentiation and cell cycle (3). Dysregulated EZH2 in tumorigenesis affects cell proliferation, apoptosis, epithelial-to-mesenchymal transition (EMT), invasion, immune response, radiotherapy, and chemotherapy resistance (3, 4). EZH2 is overexpressed and contributes to aggressive progression and poor prognosis in diverse types of human malignancies, including breast, lung, prostate, gastric, colorectal cancer, melanoma, leukemia, and lymphoma (4–7).

Nuclear SET domain-containing 2 (NSD2), also known as WHSC1 (Wolf-Hirschhorn syndrome candidate 1) or MMSET (multiple myeloma SET domain), belongs to the NSD family of histone methyltransferases. Two major transcripts of NSD2 gene are type I encoding a protein of 647 amino acids and type II encoding a protein of 1,365 amino acids (8). NSD2 specifically mediates dimethylation of lysine 36 on histone H3 (H3K36me2), a marker associated with an open conformation of chromatin and activating gene transcription (9). NSD2 was linked to oncogenesis initially because IgH enhancer drives overexpression of NSD2 in multiple myeloma with t(4,14)(p16;q32) chromosome translocation (10). Accumulating evidences have indicated that NSD2 also functions as an oncogene in several solid tumors, such as lung, colorectal, renal, cervical, prostate cancer, hepatocellular carcinoma, and osteosarcoma (11–17).

The enzymatic activity and potential therapeutic tractability of these histone methyltransferases attract broad attention in oncology, but the relationship between them is rarely understood. A previous study has shown that EZH2 may function upstream of NSD2 in prostate cancer (18). However, the relationship between EZH2 and NSD2 in BC remains unclear, and the clinicopathologic significances and prognostic values of them in BC have not been fully illuminated.

This study aimed to explore the roles and mechanisms of EZH2 and NSD2 in the pathogenesis of BC. The protein expression patterns of EZH2 and NSD2 in BC were investigated by immunohistochemistry (IHC) on tissue microarray (TMA) series and analyzing Human Protein Atlas database. The transcriptional levels of EZH2 and NSD2 in BC were evaluated using Oncomine and UALCAN databases. The correlations between their expression and clinicopathological characteristics were analyzed. Kaplan-Meier Plotter database was used to assess the prognostic values of EZH2 and NSD2 in BC. The interaction of EZH2 and NSD2 was illustrated *in vitro* in the proliferation, migration, and invasion of TNBC cells. Furthermore, bioinformatics analysis was conducted to envisage the possible functions and related pathways of EZH2/NSD2 axis in TNBC.

## Materials and Methods

### Tissue Specimen Collection

Paraffin-embedded surgical tissue specimens of 146 BC cases were collected from the Department of Pathology at First Affiliated Hospital of Dali University (Dali, China), including 126 cases of invasive ductal carcinoma (IDC), 12 cases of ductal carcinoma *in situ* (DCIS), and 8 cases of invasive lobular carcinoma (ILC). None of the patients received any preoperative adjuvant therapy. Two pathologists confirmed the pathological diagnoses of all the specimens. For controls, 24 cases of benign breast lesions were included.

### Construction of TMA

The construction of TMA followed the guidelines reported previously (19). The hematoxylin and eosin (HE) stained sections of source paraffin-embedded tissue blocks were used to identify the representative areas of the specimens by the pathologists. A TMA recipient block was constructed by a TMA instrument. A tiny core with 5 millimeters in diameter was extracted from each source tissue block and then inserted into the recipient block. Sections cut from the TMA block were used for HE staining and immunohistochemical analysis.

### HE Staining

For histological analysis under the microscope, the TMA blocks were sectioned into 4 μm and stained with HE according to a routine protocol (20).

### IHC and Protein Expression Analysis

IHC was performed on the TMA sections to assess the protein levels of EZH2 and NSD2 in BCs compared with benign breast lesions. Deparaffinization and rehydration, antigen retrieval, and endogenous peroxidase repression were performed on 4 μm-thick TMA slices. Then the slices were incubated with anti-EZH2 (1:500, Cell Signaling Technology, #5246) or anti-NSD2 (1:400, Abcam, #ab75359) antibody at 4°C overnight and then labeled using an EnVision HRP Kit (Dako) at room temperature for 30 min. The signal was visualized with diaminobenzidine-chromogen, followed by counterstaining with hematoxylin. Human Protein Atlas database (https://www.proteinatlas.org/) was further used to analyze the differential protein expression of EZH2 and NSD2 in BCs and normal breast tissues. The expression was defined using the criteria set in the database. Protein expression score is based on immunohistochemical data manually scored with regard to staining intensity (negative, weak, moderate, or strong) and fraction of stained cells (<25, 25–75, or >75%). Each combination of intensity and fractions is automatically converted into a protein expression level score as follows: negative—not detected; weak <25%—not detected; weak combined with either 25–75% or >75%—low; moderate <25%—low; moderate combined with either 25–75% or >75%—medium; strong <25%—medium, strong combined with either 25–75% or >75%—high.

### Gene mRNA Expression Analysis

Oncomine database (http://www.oncomine.org) was used to analyze the differential mRNA expression of EZH2 and NSD2 in BCs and normal breast tissues. UALCAN database (http://ualcan.path.uab.edu/) was used to analyze the correlation between EZH2 and NSD2 and the differential expression of them among different molecular subtypes of BC.

### Survival Analysis

The data of genes expression and survival information of 6,234 BC patients contained in Kaplan-Meier Plotter database were downloaded from the Gene Expression Omnibus (GEO), TCGA, and European Genome-Phenome Archive. A total of 35 datasets containing 3,951 BC patients were utilized to assess the associations of EZH2 (probe ID, 203358_s_at) or NSD2 (probe ID, 209053_s_at) mRNA expression with overall survival (OS), relapse-free survival (RFS), distant metastasis-free survival (DMFS), and postprogression survival (PPS). Hazard ratio (and 95% confidence intervals) and logrank *P*-values were calculated and displayed. The cases were categorized into high- or low-expression group using the median expression as the cutoff value.

### Cell Culture

Human TNBC cell line MDA-MB-231 was purchased from the cell bank of Institute of Zoology of the Chinese Academy of Sciences (Kunming, China) and MDA-MB-468 was purchased from Cobioer Biosciences Company (Nanjing, China). Cells were cultured in DMEM medium (Gibco) supplemented with 10% FBS (Gibco), 100 U/ml penicillin (Beyotime) and 0.1 mg/ml streptomycin (Beyotime) at 37°C with 5% CO_2_.

### Lentivirus shRNA Vectors and Stable Knockdown Cell Lines Construction

Knockdown of EZH2 or NSD2 was accomplished using specific lentivirus shRNA vectors. The target sequences of shRNA were as follows: shEZH2 (5′-GCTAGGTTAATTGGGACCAAA-3′) (21), shNSD2 (5′-GCACGCTACAACACCAAGTTT-3′) (22), and shNC (5′-TTCTCCGAACGTGTCAGGT-3′). The shRNA templates targeting EZH2 and shNC were inserted into a lentivirus shuttle vector (pGLVH1/GFP/Puro). The shRNA templates targeting NSD2 and shNC were inserted into a lentivirus shuttle vector (pGLVU6/RFP/Puro). The packing and purification of the recombinant lentivirus vectors were performed by GenePharma Company (Shanghai, China). MDA-MB-231 and MDA-MB-468 cells were infected with 5 μg/ml polybrene and the recombinant lentivirus vectors at multiplicity of infection of 20. Cells with stable knockdown of EZH2 or NSD2 were selected using 1 μg/ml puromycin.

### Adenovirus Overexpression Vector and Transient Transfection

The EZH2 adenovirus expression vector was constructed by inserting EZH2 cDNA fragment into an adenovirus shuttle vector (Adv/CMV/IRES), named Adv-EZH2. The packing and purification of the recombinant adenovirus vectors were performed by GenePharma Company (Shanghai, China). Cells were infected with recombinant adenovirus vector at a multiplicity of infection of 10.

### Quantitative Real‐Time PCR

Total RNA was extracted using a RNAprep Pure Cell Kit (Qiagen). One microgram of total RNA was used for reverse transcription using a Reverse Transcription Kit (Qiagen) following the standard protocols. QRT-PCR was subsequently performed using a Quantitative SYBR Green PCR Kit (Qiagen) on a Bio-Rad CFX96 Detection System according to the following conditions: initial step, 95˚C for 3 min; second step, 95˚C for 5 s, 60˚C for 15 s for a total of 40 cycles. The PCR primers used were following: EZH2 forward, 5′-TGCAGTTGCTTCAGTACCCATAAT-3′ and reverse, 5′-ATCCCCGTGTACTTTCCCATCATAAT-3′; NSD2 forward, 5′-ACTCCTCAAAAGACGGCAGA-3′ and reverse, 5′-TGGTGTTGTAGCGTGCTCTC-3′; CCNA2 forward, 5′- AGAAACAGCCAGACATCACTAA-3′ and reverse, 5′- TTCAAACTTTGAGGCTAACAGC-3′; CDK2 forward, 5′- CCTGGGCTGCAAATATTATTCC-3′ and reverse, 5′- TGGCTTGTAATCAGGCATAGAA-3′; KDM2B forward, 5′- TTAAGATGCCTGACCCTGATTT-3′ and reverse, 5′- CTCTAGGCTGATGACGTTGTAC-3′; KIF11 forward, 5′- CATACTCTAGTCGTTCCCACTC-3′ and reverse, 5′- CAACCAAGTTCAACTTTCCGAT-3′; KIF23 forward, 5′- AAGAGGATCATTGCGGCAGGTTAC-3′ and reverse, 5′- TTGCATGTTAGAGGCGGGCTTATG-3′; PCNA forward, 5′- TAATTTCCTGTGCAAAAGACGG-3′ and reverse, 5′- AAGAAGTTCAGGTACCTCAGTG-3′; GAPDH forward, 5′-TGCACCACCAACTGCTTAGC-3′ and reverse, 5′-GGCATGGACTGTGGTCATGAG-3′. The mRNA expression were calculated and normalized using the 2^−ΔΔCt^ method relative to GAPDH. The relative expression value of the group with lower expression was defined as 1. All experiments were performed in triplicate.

### Western Blot

Cells were lysed with RIPA lysis buffer (Cwbio) supplemented with a protease inhibitor cocktail (Cwbio), and the concentration of protein was determined using the BCA Protein Assay Kit (Cwbio). Thirty micrograms of protein was separated by sodium dodecyl sulfate-polyacrylamide gel electrophoresis (SDS‐PAGE) and transferred onto PVDF membranes (Millipore), followed by incubating with 5% non‐fat milk for 2 h at room temperature. The membranes were incubated with specific primary antibodies against EZH2 (1:1,000, Cell Signaling Technology #5246), NSD2 (1:1,000, Abcam, #ab75359), H3K27me3 (1:1,000, Cell Signaling Technology #9733), H3K36me2 (1:1,000, Cell Signaling Technology #2901), and β-actin (1:1,000, Cwbio #CW0096) at 4°C overnight. The membranes were further washed and incubated with secondary antibody (1:2,000, Cwbio) at room temperature for 1h. Chemiluminescence was detected using an ECL Immobilon Western Kit (Millipore), followed by exposure in ChemiDoc™ MP Imaging System (Bio-Rad).

### CCK8 Assay

The proliferation ability of cells was measured using a CCK8 Assay Kit (Beyotime) according to the manufacturer’s protocols. Briefly, 4×10^3^ stably transfected or transient transfected cells were seeded into 96-well plates and CCK8 assay was performed at different time points of 0, 24, 48, 72, and 96 h. Ten microliters of CCK8 reagent was added into each well and incubated for 1 h. Subsequently, the absorbance at 450 nm wavelength was measured on a microplate spectrophotometer (Gene Company).

### Wound Healing Assay

Wound healing assay was performed to observe the migration ability of cells. 7x10^5^ cells were plated in six−well plates and a 10 μl pipette tip was used to scratch a vertical wound. The detached cells were removed and the adherent cells were incubated with serum-free medium. Images of the scratched fields (100×) were captured at 0 and 24 h using a light microscope (Olympus BX53), and the area of the wound was measured with ImageJ software. At least three scratched fields were recorded in each group.

### Transwell Assay

Transwell chambers (Corning) with 8 µm pores were used to evaluate the invasion ability of cells. 2×10^5^ cells in serum-free medium were seeded into the upper chamber with Matrigel (BD Biosciences, #356234). Medium containing 10% FBS was added to the lower chambers. After 8 h, the cells remaining on the upper membrane were removed, while the cells invading through the membrane were fixed with paraformaldehyde, stained with crystal violet (Beyotime), and observed under a light microscope (200×). 33% acetic acid solution was used to elute the staining and the absorbance at 570 nm wavelength was measured on a microplate spectrophotometer (Gene Company).

### Protein-Protein Interaction Network Construction and Functional Enrichment Analysis

The co-expressed genes of EZH2 and NSD2 with a Pearson correlation (≥0.5) in BC were analyzed using UALCAN database. STRING online system (https://string-db.org/) was used to construct a protein-protein interaction (PPI) interactions network of EZH2, NSD2 and their co-expressed genes. Subsequently, these genes were subjected to gene ontology (GO) and Kyoto Encyclopedia of Genes and Genomes (KEGG) enrichment analysis in Database for Annotation, Visualization and Integrated Discovery (DAVID) (https://david.ncifcrf.gov/home.jsp). *P* < 0.05 was set as the cutoff value.

### Statistical Analysis

Statistical analysis was performed using SPSS 21.0 software and datas were expressed as the mean ± standard deviation (SD). Differences between two groups were assessed by Student’s t test. The chi-square test was used to determine differences of EZH2 or NSD2 expression among groups with different clinicopathological characteristics. The spearman rank correlation test was used to analyze the expression correlation between EZH2 and NSD2. Kaplan-Meier analysis followed by the logrank test was used to analyze the association between EZH2 or NSD2 expression and the survival rate. *P* < 0.05 indicated a statistically significant difference.

## Results

### Construction of TMA Containing BCs and Benign Lesions

**Figure 1:**
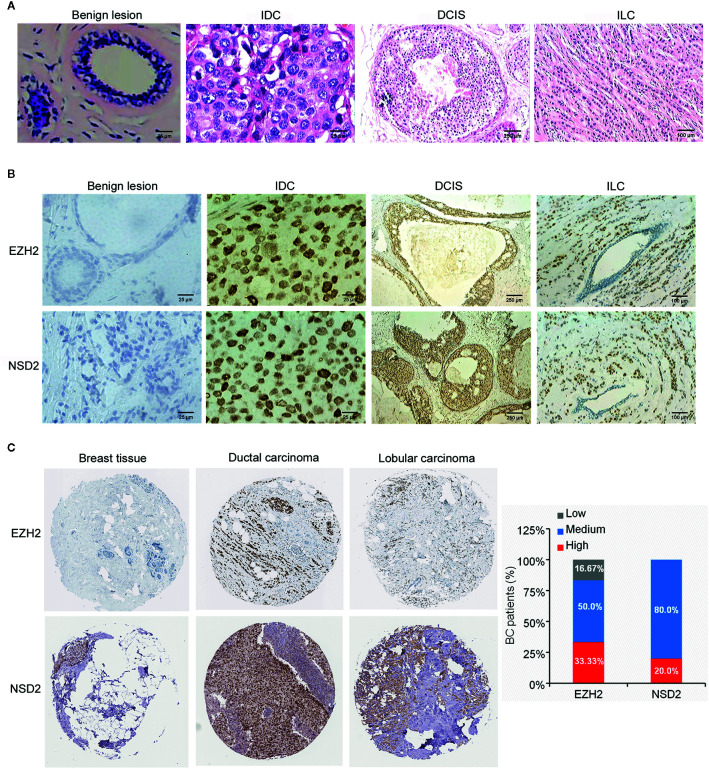
EZH2 and NSD2 proteins were overexpressed in breast cancer (BC) tissues. **(A)** HE staining of tissue microarray (TMA) sections containing benign lesion (×400), IDC (×400), DCIS (×40), and ILC (×100). **(B)** EZH2 and NSD2 expression in benign lesion (×400), IDC (×400), DCIS (×40), and ILC (×100) tissues were analyzed by IHC. **(C)** EZH2 and NSD2 expression in normal breast tissue, ductal carcinoma and lobular carcinoma were analyzed using Human Protein Atlas database.

Firstly, the TMA blocks were constructed using paraffin-embedded surgical tissue specimens composed of 24 cases of benign breast lesions and 146 cases of BC, including 126 cases of IDC, 12 cases of DCIS, and 8 cases of ILC. HE staining showed the structural integrity and histological features of core lesion regions in TMA sections. The microscopic appearance of IDC was heterogeneous, ranging from tumors with low-grade nuclei and well-developed tubule formation to tumors consisting of sheets of anaplastic cells (**Figure 1A**). IDC cells invaded into the stroma with a surrounding desmoplastic response (**Figure 1A**). DCIS cells were confined to the duct and the nuclear appearance ranged from bland or monotonous to pleomorphic, whereas the basement membrane all remains intact (**Figure 1A**). ILC cells usually aligned in chains or strands and invaded into stroma individually (**Figure 1A**).

### EZH2 and NSD2 Are Coordinately Overexpressed in BC Tissues

To investigate the protein expression patterns of EZH2 and NSD2 in BC, IHC assay was performed in TMA sections. Both EZH2 and NSD2 showed a nuclear staining pattern (**Figure 1B**). The protein expression levels of EZH2 and NSD2 in benign lesions were significantly lower than that in cancers (**Figure 1B** and **Table 1**). EZH2 expression displayed strong correlation with NSD2 expression in all BC samples (Spearman correlation coefficient *r_s_* = 0.694, *P* < 0.001, **Table 2**). In different histological types of samples, EZH2 expression was strongly correlated with NSD2 expression in IDC (Spearman correlation coefficient *r_s_* = 0.745, *P* < 0.001) and DCIS (Spearman correlation coefficient *r_s_* = 0.683, *P* < 0.05) (**Table 2**). However, there was no correlation between their expression in ILC probably due to the limited samples (Spearman correlation coefficient *r_s_* = −0.143, *P* > 0.05, **Table 2**). Besides, analysis using Human Protein Atlas database also revealed that EZH2 and NSD2 were predominantly located in the cell nucleus, and the medium/high expression rates of EZH2 and NSD2 in BC were 83.33% (10/12 cases) and 100% (10/10 cases), respectively (**Figure 1C**).

**Table 1:** EZH2 and NSD2 protein expression in breast cancer (BC) and benign lesion tissues.

Tissue sample	n	EZH2 expression		*P*-value	NSD2 expression		*P-*value
		Positive (%)	Negative (%)		Positive (%)	Negative (%)	
Benign lesion	24	4 (16.7)	20 (83.3)	<0.001^***^	3 (12.5)	21 (87.5)	<0.001^***^
BC	146	120 (82.2)	26 (17.8)		116 (79.5)	30 (20.5)	

***P < 0.001.

**Table 2:** The protein expression levels of EZH2 and NSD2 were correlated in breast cancer (BC) tissues.

	EZH2 expression	n	NSD2 expression		*r_s_*	*P-*value
			Positive	Negative		
BC	Positive	120	111	9	0.694	<0.001^***^
	Negative	26	5	21		
IDC	Positive	106	98	8	0.745	<0.001^***^
	Negative	20	2	18		
DCIS	Positive	7	7	0	0.683	0.014^*^
	Negative	5	2	3		
ILC	Positive	7	6	1	−0.143	0.736
	Negative	1	1	0		

*P < 0.05, ***P < 0.001.

**Figure 2:**
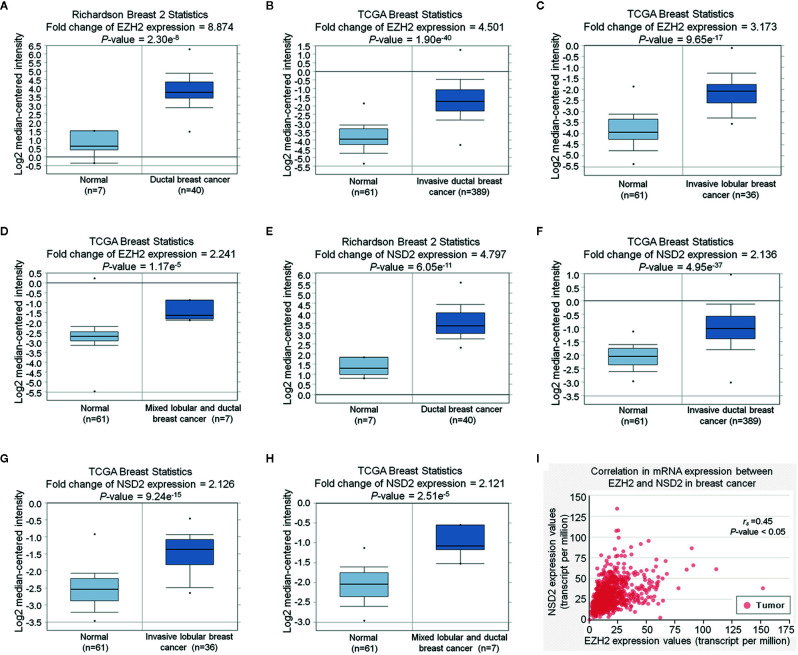
EZH2 and NSD2 messenger RNA (mRNA) levels were upregulated in breast cancer (BC) tissues. **(A–D)** The relative expression of EZH2 mRNA in BC and normal tissues was analyzed using Oncomine database. **(E–H)** The relative expression of NSD2 mRNA in BC and normal tissues was analyzed using Oncomine database. **(I)** The correlation between EZH2 and NSD2 mRNA expression in BC was analyzed using UALCAN database.

To examine the mRNA expression of EZH2 and NSD2 in breast cancer, Oncomine database was used to analyze their mRNA expression. According to four different studies, the mRNA expression level of EZH2 was upregulated in BC by 2.241 to 8.874 folds relative to that in normal breast tissues (**Figures 2A–D**). Meanwhile, the mRNA expression level of NSD2 was increased in BC by 2.121 to 4.797 folds (**Figures 2E–H**). A positive correlation between EZH2 and NSD2 mRNA expression in BC was also demonstrated using UALCAN database (Spearman correlation coefficient *r_s_* = 0.45, *P* < 0.05, **Figure 2I**).

### High Expression of EZH2 and NSD2 Are Associated with Poor Prognosis of BC

**Figure 3:**
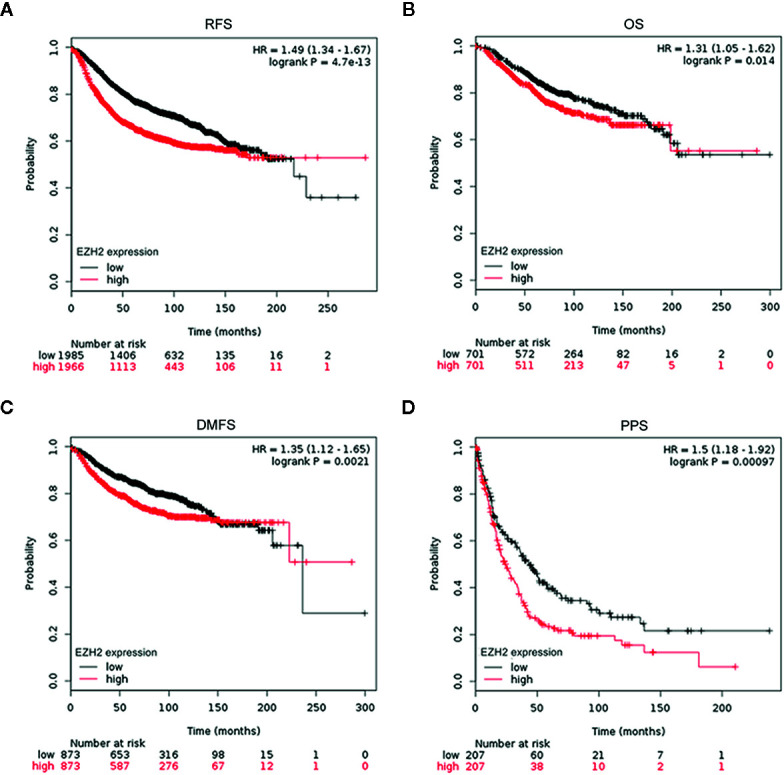
Survival curves for relapse-free survival (RFS) **(A)**, overall survival (OS) **(B)**, distant metastasis-free survival (DMFS) **(C)**, and postprogression survival (PPS) **(D)** in patients with BC according to EZH2 expression were analyzed using Kaplan-Meier Plotter database.

**Figure 4:**
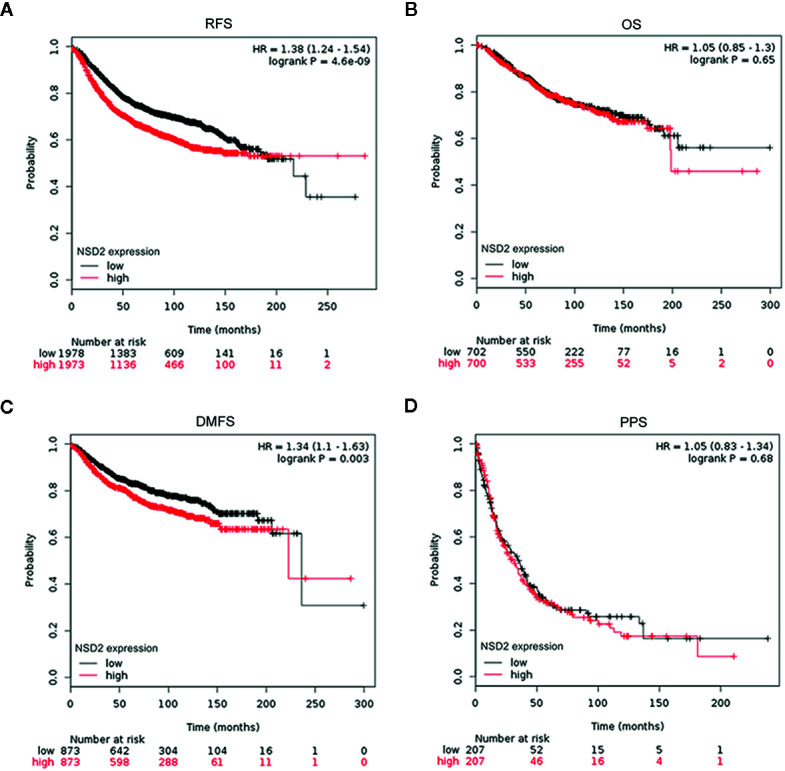
Survival curves for relapse-free survival (RFS) **(A)**, overall survival (OS) **(B)**, distant metastasis-free survival (DMFS) **(C)**, and postprogression survival (PPS) **(D)** in patients with breast cancer (BC) according to NSD2 expression were analyzed using Kaplan-Meier Plotter database.

To evaluate the potential roles of EZH2 and NSD2 in BC, the relationship between EZH2 or NSD2 protein expression and the clinicopathological characteristics of BC patients was analyzed. As presented in **Table 3**, both EZH2 and NSD2 protein expression correlated with pathological grade of tumor and lymph node metastasis; the high grade and positive lymph node involvement were associated with increased rate of positive EZH2 and NSD2 (*P* < 0.001, *P* = 0.003). On the contrary, EZH2 and NSD2 protein expression were not associated with other clinicopathological parameters, including age, size of tumor and histological type of tumor. Kaplan-Meier Plotter database was used to investigate the relationship between EZH2 or NSD2 and survival rate of BC patients. 3,951 patients were classified into high- or low-expression group according to the median expression of EZH2 or NSD2. As shown in **Figure 3**, high EZH2 expression level was significantly correlated with lower RFS (*P* = 4.17e−13, **Figure 3A**), OS (*P* = 0.014, **Figure 3B**), DMFS (*P* = 0.0021, **Figure 3C**) and PPS (*P* = 0.00097, **Figure 3D**) rates. High NSD2 expression was associated with RFS (*P* = 4.6e−9, **Figure 4A**) and DMFS (*P* = 0.003, **Figure 4C**), but had no effect on OS (*P* = 0.65, **Figure 4B**) and PPS (*P* = 0.68, **Figure 4D**) in BC patients. By evaluating risk factors associated with patients using Cox proportional hazard models, it was determined that EZH2 and NSD2 were significant predictors of outcomes in BC patients (**Figures 3** and **4**).

**Table 3:** The correlation between EZH2 or NSD2 expression and clinicopathological characteristics of breast cancer (BC).

Characteristics	n	EZH2 expression		*P*-value	NSD2 expression		*P-*value
		Positive (%)	Negative (%)		Positive (%)	Negative (%)	
Age (years)				0.09			0.331
≤50	91	71 (78.0)	20 (22.0)		70 (76.9)	21 (23.1)	
>50	55	49 (89.1)	6 (10.9)		46 (83.6)	9 (16.4)	
Tumor size (cm)				0.189			0.884
≤2	38	29 (76.3)	9 (23.7)		30 (78.9)	8 (21.1)	
>2 ~ ≤5	79	64 (81.0)	15 (19.0)		62 (78.5)	17 (21.5)	
>5	29	27 (93.1)	2 (6.9)		24 (82.8)	5 (17.2)	
Histological type				0.076			0.459
IDC	126	106 (84.1)	20 (15.9)		101 (80.2)	25 (19.8)	
DCIS	12	7 (58.3)	5 (41.7)		8 (66.7)	4 (33.3)	
ILC	8	7 (87.5)	1 (12.5)		7 (87.5)	1 (12.5)	
Pathological grade				<0.001^***^			<0.001^***^
I	43	26 (60.5)	17 (39.5)		22 (51.2)	21 (48.8)	
II	81	72 (88.9)	9 (11.1)		74 (91.4)	7 (8.6)	
III	22	22 (100.0)	0 (0.0)		20 (90.9)	2 (9.1)	
Lymph node metastasis				<0.001^***^			0.003^**^
No	72	50 (69.4)	22 (30.6)		50 (69.4)	22 (30.6)	
Yes	74	70 (94.6)	4 (5.4)		66 (89.2)	8 (10.8)	

**P < 0.01, ***P < 0.001.

### EZH2 and NSD2 Expression Are Higher in TNBC Than That in Other Molecular Subtypes of BC

**Figure 5:**
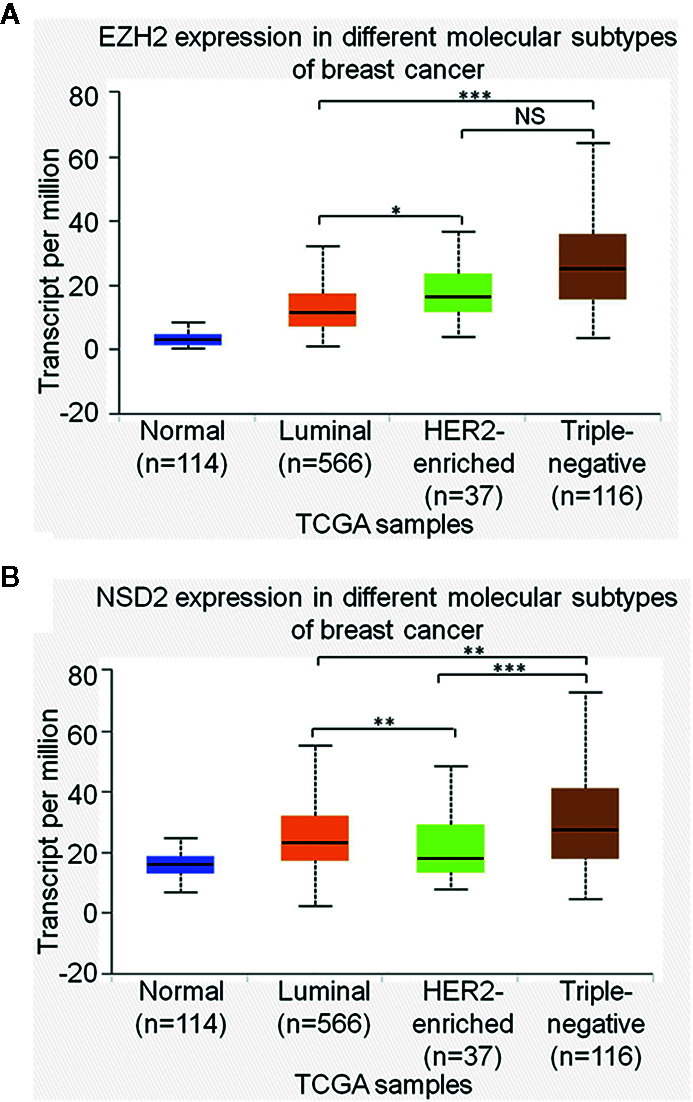
The relative expression of EZH2 messenger RNA (mRNA) **(A)** and NSD2 mRNA **(B)** among different molecular subtypes of breast cancer (BC) were analyzed using UALCAN database. *P < 0.05, **P < 0.01, ***P < 0.001.

Interestingly, we observed that the positive rates of EZH2 and NSD2 protein expression in TNBC were higher than that in other molecular subtypes of BC, including luminal A, luminal B and HER2-enriched BC (*P* = 0.033, *P* = 0.026, **Table 4**). UALCAN database was used to explore the transcriptional levels of EZH2 and NSD2 in different molecular subtypes of BC. EZH2 expression in TNBC was significantly higher than that in luminal BC (*P* = 8.88e−16), while there was no statistical difference in EZH2 expression between TNBC and HER2-enriched BC (*P* = 0.103, **Figure 5A**). Compared with luminal and HER2-enriched BC, NSD2 expression was higher in TNBC (*P* = 8.83e−3, *P* = 8.04e−5, **Figure 5B**).

**Table 4:** The correlation between EZH2 or NSD2 expression and molecular subtypes of breast cancer (BC).

Molecular subtypes	n	EZH2 expression		*P*-value	NSD2 expression		*P-*value
		Positive (%)	Negative (%)		Positive (%)	Negative (%)	
Luminal A	69	56 (81.2)	13 (18.8)	0.033^*^	55 (79.7)	14 (20.3)	0.026^*^
Luminal B	32	23 (71.9)	9 (28.1)		22 (68.8)	10 (31.2)	
HER2-enriched	10	7 (70.0)	3 (30.0)		6 (60.0)	4 (40.0)	
Triple-negative	35	34 (97.1)	1 (2.9)		33 (94.3)	2 (5.7)	

*P < 0.05.

### EZH2 Upregulates NSD2 Expression and Histone Methylation and Promotes the Proliferation, Migration, and Invasion of TNBC Cells

**Figure 6:**
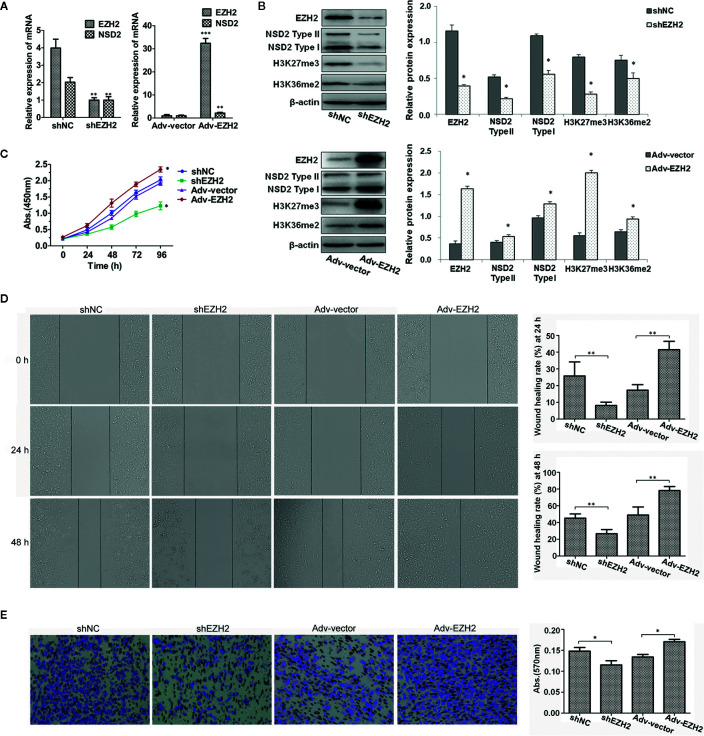
Stable knockdown of EZH2 inhibited the proliferation, migration and invasion of MDA−MB−231 cells, whereas overexpression of EZH2 displayed an inverse phenotype. Stable knockdown or transient overexpression of EZH2 was performed in MDA-MB-231 cells. **(A)** The relative messenger RNA (mRNA) levels of EZH2 and NSD2 were determined by qRT-PCR. **(B)** The protein levels of EZH2, NSD2 and histone methylation markers were determined by Western blot. Left panel showed the representative images of protein expression and right panel showed the fold changes of protein levels. **(C)** The cell proliferation ability was determined by CCK8 assay. **(D)** The cell migration ability was determined by wound healing assay. Left panel showed the representative images of cell migration and right panel showed the wound healing rate at 24 and 48 h. **(E)** The cell invasion ability was determined by transwell assay. Left panel showed the representative images of cell invasion and right panel showed the absorbance of invasive cells stained by crystal violet. *P < 0.05, **P < 0.01, ***P < 0.001.

As EZH2 and NSD2 expression were significantly upregulated in TNBC, a TNBC cell line MDA-MB-231 was used to examine the functional relationship between EZH2 and NSD2. We constructed a MDA-MB-231 cell line in which EZH2 expression was stably knocked down by lentivirus shRNA vector. The results showed that knockdown of EZH2 decreased NSD2 mRNA and protein expression remarkably in MDA-MB-231 cells (**Figures 6A, B**). In addition, knockdown of EZH2 also reduced the levels of H3K27me3 and H3K36me2, which are histone methylation markers catalyzed by EZH2 and NSD2, respectively (**Figure 6B**). By contrast, adenovirus-mediated EZH2 overexpression significantly increased NSD2 expression as well as the methylation levels of H3K27 and H3K36 in MDA-MB-231 cells (**Figures 6A, B**). These results suggested that NSD2 functions as a downstream gene of EZH2. To assess the biological role of EZH2 in TNBC, CCK8 assay revealed that cell proliferation was significantly impaired in shEZH2 stably transfected cells (**Figure 6C**). Next, we evaluated cancer cell migration and invasion abilities through wound healing assay and transwell assay. Decreased expression of EZH2 also impeded the migration and invasion of cells (**Figures 6D, E**). On the contrary, overexpression of EZH2 in MDA-MB-231 cells displayed an inverse phenotype (**Figures 6C–E**). Similar effects were observed in MDA-MB-468 cells with knockdown or overexpression of EZH2 (**Supplementary Figures 1A–E**).

### EZH2-Mediated Oncogenic Effects Require NSD2 Expression

**Figure 7:**
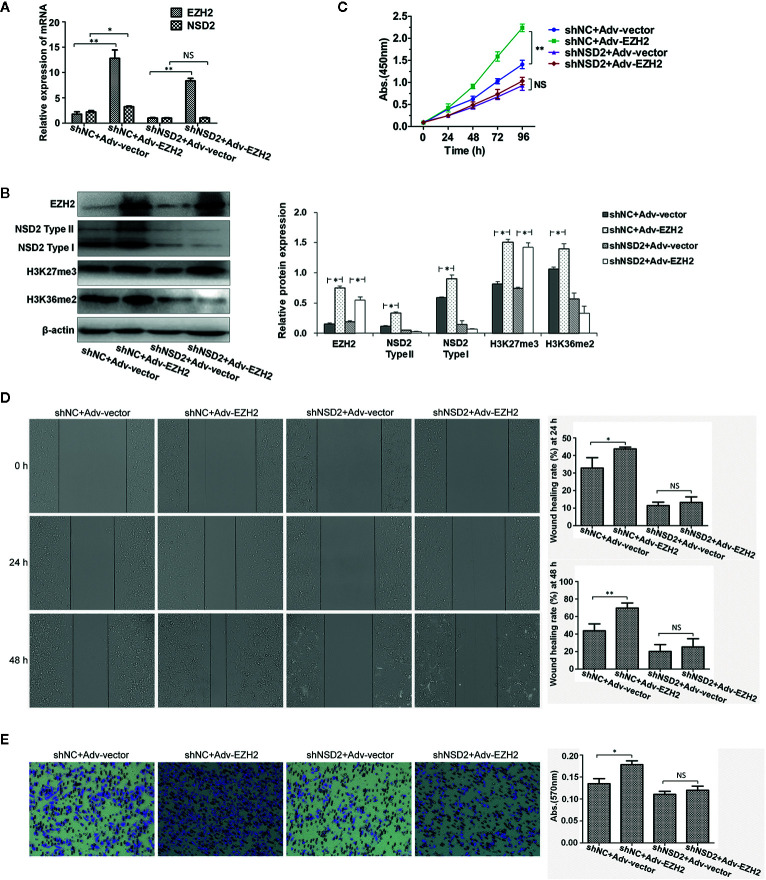
Knockdown of NSD2 attenuated the proliferation, migration and invasion abilities of EZH2-overexpressing MDA-MB-231 cells. Transient overexpression of EZH2 was performed in MDA-MB-231 cells with stable knockdown of NSD2. **(A)** The relative messenger RNA (mRNA) levels of EZH2 and NSD2 were determined by qRT-PCR. **(B)** The protein levels of EZH2, NSD2, and histone methylation markers were determined by Western blot. Left panel showed the representative images of protein expression and right panel showed the fold changes of protein levels. **(C)** The cell proliferation ability was determined by CCK8 assay. **(D)** The cell migration ability was determined by wound healing assay. Left panel showed the representative images of cell migration and right panel showed the wound healing rate at 24 and 48 h. **(E)** The cell invasion ability was determined by transwell assay. Left panel showed the representative images of cell invasion and right panel showed the absorbance of invasive cells stained by crystal violet. *P < 0.05, **P < 0.01, NS, non-significant.

Knockdown of NSD2 could inhibit the proliferation, migration, and invasion in MDA-MB-231 and MDA-MB-468 cells (**Supplementary Figures 2A–D**). To determine the role of NSD2 in EZH2-mediated oncogenic effects, overexpression of EZH2 was performed in MDA-MB-231 cells with stable knockdown of NSD2 and control cells. As expected, overexpression of EZH2 upregulated H3K27me3 level in cells, but EZH2-induced NSD2 expression and H3K36me2 level were abolished by NSD2 shRNA treatment (**Figures 7A, B**). Cells overexpressing EZH2 displayed high proliferation, migration, and invasion abilities, while all of these were dramatically attenuated upon NSD2 knockdown, suggesting that NSD2 is a critical downstream mediator for EZH2-regulated cell phenotypes (**Figures 7C–E**). Overexpression of EZH2 in MDA-MB-468 cells with knockdown of NSD2 showed the similar effects with that in MDA-MB-231 cells (**Supplementary Figures 3A–E**).

### Function and Pathway Enrichment Analysis of Co-Expressed Genes of EZH2/NSD2 Axis

**Figure 8:**
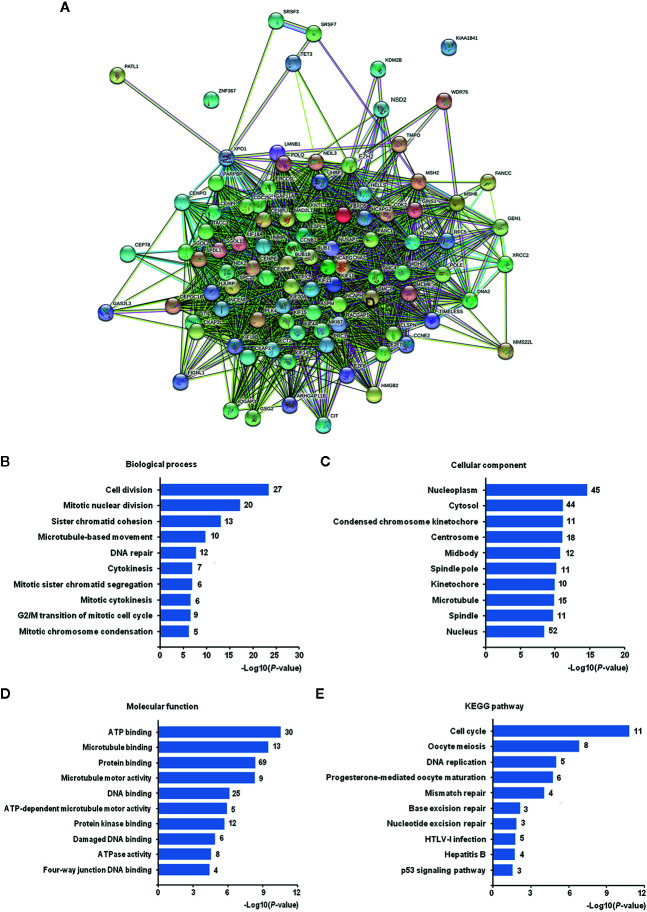
The Gene Ontology (GO) and Kyoto Encyclopedia of Genes and Genomes (KEGG) pathway enrichment analysis of co-expressed genes of EZH2/NSD2 axis. **(A)** The protein-protein interaction (PPI) network of EZH2, NSD2, and 94 co-expressed genes in breast cancer (BC). **(B)** Top 10 of enriched biological processes. **(C)** Top 10 of enriched cellular components. **(D)** Top 10 of enriched molecular functions. **(E)** Top 10 of enriched KEGG pathways.

To analyze the functions and pathways that EZH2/NSD2 axis may be involved in, 94 co-expressed genes of EZH2 and NSD2 with a Pearson correlation (≥0.5) in BC were obtained using UALCAN database (**Table S1**). A PPI network comprising 96 nodes and 2,087 edges with PPI-enrichment *P*-value < 1.0e−16 was constructed using STRING online system to explore the interactions of EZH2, NSD2, and their co-expressed genes (**Figure 8A**). GO enrichment analysis revealed that these genes were significantly enriched in the biological processes (BP) about cell proliferation, such as cell division, mitotic nuclear division, chromosome segregation and condensation, microtubule-based movement, cytokinesis, transition of mitotic cell cycle, and DNA repair (**Figure 8B** and **Table S2**). These genes were widely distributed in various kinds of cellular component (CC) and mostly located in nucleus, nucleoplasm and cytosol (**Figure 8C** and **Table S3**). In terms of molecular function (MF), these genes were responsible for ATP, microtubule, DNA, and protein binding, thus maintaining the activity of ATPase, microtubule motor and protein kinase (**Figure 8D** and **Table S4**). KEGG pathway analysis showed that these genes were mainly enriched in the pathways involved in cell cycle, meiosis, DNA replication, and repair (**Figure 8E** and **Table S5**).

### Function and Pathway Enrichment Analysis of Interactive Genes of EZH2/NSD2 Axis

**Figure 9:**
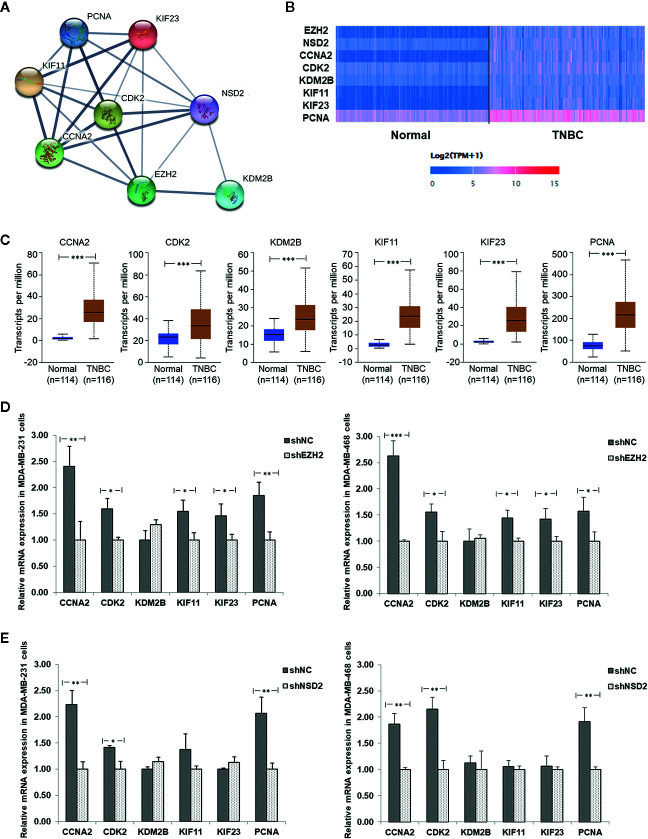
The protein-protein interaction (PPI) network and relative expression of interactive genes of EZH2/NSD2 axis in triple-negative breast cancer (TNBC). **(A)** The PPI network of EZH2, NSD2 and the interactive genes in BC. **(B)** The expression heatmap of EZH2, NSD2, and the interactive genes in TNBC. **(C)** The median expression levels of CCNA2, CDK2, KDM2B, KIF11, KIF23, and PCNA in TNBC and normal breast tissues were analyzed using UALCAN database. **(D)** The relative mRNA levels of CCNA2, CDK2, KDM2B, KIF11, KIF23, and PCNA were analyzed by qRT-PCR in MDA-MB-231 and MDA-MB-468 cells with stable knockdown of EHZ2. **(E)** The relative mRNA levels of CCNA2, CDK2, KDM2B, KIF11, KIF23, and PCNA were analyzed by qRT-PCR in MDA-MB-231 and MDA-MB-468 cells with stable knockdown of NSD2. *P<0.05, **P<0.01, ***P<0.001.

Based on the PPI analysis (**Figure 8A**), 45 genes interacted with EZH2 and seven genes interacted with NSD2. Six genes interacting with both EZH2 and NSD2 were further identified, that were cyclin A2 (CCNA2), cyclin dependent kinase 2 (CDK2), lysine demethylase 2B (KDM2B), kinesin family member 11 (KIF11), kinesin family member 23 (KIF23), and proliferating cell nuclear antigen (PCNA) (**Figure 9A**). The eight genes in **Figure 9A** were subjected to GO and KEGG pathway analysis. As shown in **Table 5**, numerous biological processes were associated with the development of cancer, including mitotic nuclear division, cell division, cellular response to multiple stimuli, Ras protein signal transduction, and transition of mitotic cell cycle. The significant enriched GO terms in CC were nucleoplasm, cyclin A2-CDK2 complex, centrosome, nucleus, kinesin complex, and spindle (**Table 5**). MF analysis indicated that these genes may function in RNA polymerase II core promoter sequence-specific DNA binding and microtubule motor activity (**Table 5**). The enriched KEGG pathway of these genes is cell cycle pathway (**Table 5**). The expression of CCNA2, CDK2, KDM2B, KIF11, KIF23, and PCNA in 116 cases of TNBC and 114 cases of normal breast tissue were evaluated using UALCAN database. Similar with the expression patterns of EZH2 and NSD2, the gene expression heatmap showed that these six genes were also significantly upregulated in TNBC (**Figure 9B**). Compared with normal breast tissues, the median expression levels of CCNA2, CDK2, KDM2B, KIF11, KIF23, and PCNA in TNBC were increased by 14.5, 1.44, 1.55, 9.29, 12.47, and 2.87 folds, respectively (*P*<0.001, **Figure 9C**). Additionally, the expression levels of these genes were further verified by qRT-PCR in MDA-MB-231 and MDA-MB-468 cells with stable knockdown of EZH2 or NSD2. Knockdown of EZH2 decreased the expression of CCNA2, CDK2, KIF11, KIF23, and PCNA, but had no effect on the expression of KDM2B (**Figure 9D**). The expression levels of CCNA2, CDK2, and PCNA were also downregulated in cells with stable knockdown of NSD2, while no significant changes of KDM2B, KIF11and KIF23 expression were observed (**Figure 9E**).

**Table 5:** Function and pathway enrichment analysis of interactive genes of EZH2/NSD2 axis.

Category	Term	Description	*P-*value
BP term	GO:0007067	Mitotic nuclear division	0.0031
BP term	GO:0071732	Cellular response to nitric oxide	0.0050
BP term	GO:0051301	Cell division	0.0061
BP term	GO:0097421	Liver regeneration	0.0103
BP term	GO:0070301	Cellular response to hydrogen peroxide	0.0202
BP term	GO:0006977	DNA damage response, signal transduction by p53 class mediator resulting in cell cycle arrest	0.0220
BP term	GO:0007265	Ras protein signal transduction	0.0248
BP term	GO:0007018	Microtubule-based movement	0.0286
BP term	GO:0006890	Retrograde vesicle-mediated transport, Golgi to ER	0.0289
BP term	GO:0032355	Response to estradiol	0.0321
BP term	GO:0019886	Antigen processing and presentation of exogenous peptide antigen *via* MHC class II	0.0324
BP term	GO:0000082	G1/S transition of mitotic cell cycle	0.0359
CC term	GO:0005654	Nucleoplasm	4.34E-04
CC term	GO:0097124	Cyclin A2-CDK2 complex	6.58E-04
CC term	GO:0005813	Centrosome	0.0077
CC term	GO:0005634	Nucleus	0.0104
CC term	GO:0005871	Kinesin complex	0.0173
CC term	GO:0005819	Spindle	0.0392
MF term	GO:0000979	RNA polymerase II core promoter sequence-specific DNA binding	0.0201
MF term	GO:0003777	Microtubule motor activity	0.0281
KEGG pathway	hsa04110	Cell cycle	0.0019

## Discussion

Generally, breast carcinogenesis arises from altered gene expression or gene mutations, which eventually leads to the dysregulation of numerous oncogenes, tumor suppressor gene, and non-coding RNAs. Epigenetic alterations are heritable changes which affect gene expression profiles but do not change the primary DNA sequence (23). Investigations about aberrant epigenetic factors including histone modifications and DNA methylation, mainly focus on the molecular mechanisms in cancer initiation and development, the new biomarkers for cancer progression and the potential of epigenetic therapy for BC (23). Histone methyltransferases are potential therapeutic targets for malignancies, but it is poorly understood that how they are linked together. Asangani (18) analyzed the transcriptional expression of EZH2 and 51 histone methyltransferases in several published and unpublished RNAseq data sets, which comprised 474 malignant and benign samples, representing 14 different tissue types. In all the histone methyltransferases, NSD2 displayed the strongest correlation with EZH2 expression (Spearman correlation coefficient r = 0.79, *P* = 7.27e−99) (18). Similarly, a meta-analysis of 1,755 samples from 22 published microarray gene expression studies in 15 different cancers using Oncomine database also revealed that NSD2 displayed the most correlated transcriptional expression with EZH2 in a majority of studies (82%) (18).

Studies have highlighted the multiple roles of EZH2 in the pathogenesis of BC. The increased expression of EZH2 in histologically normal breast epithelium is associated with higher risk of developing cancer, suggesting that EZH2 may be a potential marker for identifying preneoplastic lesions of the breast as well as a possible target for preventative intervention (24). Previous studies showed that EZH2 is frequently expressed in 75.7% of inflammatory BCs and associated with unfavorable prognostic factors including larger size, high histological grade, increased risk for distant metastasis, and first degree family history of BC (25, 26). Therefore, EZH2 may function as a useful biomarker of long-term metastatic risk and worse prognosis in women with BC, and warrant further validation studies. As a novel oncogene identified in recent years, NSD2 overexpression is associated with the clinical stage and TNM classification in BC (27). Gene expression profiling analysis revealed that NSD2 expression is highly elevated in endocrinotherapy-resistant BC tissues and cell lines (28). Consistent with previous reports, this study demonstrated that EZH2 and NSD2 proteins were expressed in 82.2% (120/146) and 79.5% (116/146) cases of BC, respectively. Gene mRNA expression analysis also showed that the transcriptional levels of EZH2 and NSD2 were upregulated in BC. Moreover, we found a high correlation between the expression of these two histone methyltransferases at both protein and mRNA levels. Both EZH2 and NSD2 expression strongly correlated with histological grade of tumor and lymph node metastasis. Although the effect of EZH2 or NSD2 on survival in BC patients has been previously described, this study is the first to offer a comprehensive analysis about the prognostic values of EZH2 and NSD2 for RFS, OS, DMFS, and PPS in a large group of BC patients. Increased NSD2 expression level was correlated with shorter RFS and DMFS, and EZH2 could be used for prognosis assessment in RFS, OS, DMFS and PPS. Notably, this study highlighted that EZH2 and NSD2 were highly expressed in TNBC when compared with other molecular subtypes of BC. These results implied that EZH2 and NSD2 are involved in the development and progression of BC, especially for TNBC.

Ectopic overexpression of EZH2 induces malignant transformation of the mammary gland cells by promoting cell invasion and anchorage-independent growth *in vitro* (29). EZH2 overexpression disrupts ductal morphogenesis and causes epithelial hyperplasia *in vivo* (30). In this study, knockdown of EZH2 significantly inhibited TNBC cell proliferation and impaired cell migration and invasion, whereas overexpression of EZH2 produced an inverse phenotype. Silencing endogenous expression of NSD2 significantly suppresses the proliferation and metastasis of BC cells through inhibiting the Wnt/β-catenin signaling pathway (27). NSD2 induces resistance to tamoxifen in BC by methylating H3K36me2 at the promoters of key glucose metabolic enzyme genes and heightening the pentose phosphate pathway (28). NSD2 also mediates TNBC cell resistance to the EGFR inhibitor gefitinib *via* stimulating ADAM9-EGFR-AKT signaling (31). This is the first report that knockdown of NSD2 abolishes EZH2-mediated TNBC cell proliferation, migration and invasion, indicating that the oncogenic function of EZH2 depends on NSD2 expression.

A previous report suggested that the regulation of NSD2 expression by EZH2 may occur at the posttranscriptional level through microRNAs network (18). The authors found that a significant induction of NSD2 protein in normal epithelial cells when EZH2 is overexpressed, but NSD2 transcription level remains unchanged (18). In prostate cancer cells, Asangani (18) found that EZH2 catalyzes H3K27 trimethylation to inhibit miR-26a, miR-31, and miR-203, which target the 3’-UTR of NSD2. However, we testified that EZH2 functioned upstream of NSD2 and upregulated both the mRNA and protein expression of NSD2, as well as H3K27me3 and H3K36me2 levels in TNBC cells. It was reported that EZH2 has a dual nature, because it can act as a gene repressor or activator (3). On one hand, EZH2 can restrain gene expression by methylating both histone and non-histone proteins on promoter regions of several genes, such as CDKN1C, FOXC1, E-cadherin, RAD51, and RUNX3 (3, 32). On the other hand, it can activate gene expression and pathways by interacting with transcription factors and co-factors, such as androgen receptor, β-catenin, STAT3, estrogen receptor α, RelA/RelB, and PCNA-associated factor (3). Based on the results of the present study, we speculated that EZH2 may enhance the transcription of NSD2 though interacting with some transcription factors or co-factors in TNBC. Thus, further researches will be needed to elucidate the direct or indirect regulation of NSD2 expression by EZH2.

**Figure 10:**
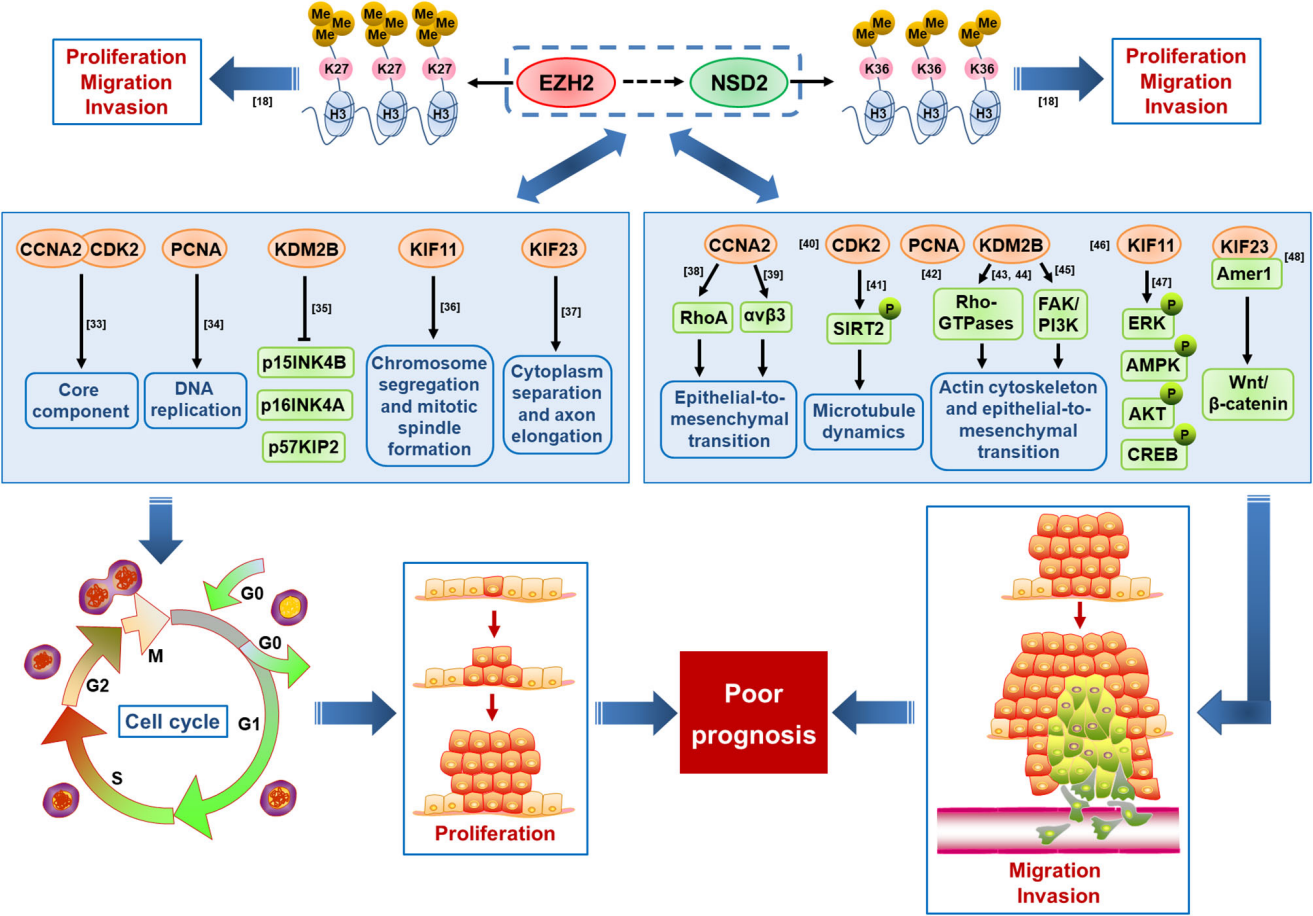
A schematic diagram displaying the proposed mechanism by which EZH2/NSD2 axis promotes cell proliferation, migration and invasion and mediates poor prognosis in triple-negative breast cancer (TNBC).

In 94 co-expressed genes with EZH2 and NSD2, six interactive genes with EZH2/NSD2 axis were further identified, that are CCNA2, CDK2, KDM2B, KIF11, KIF23, and PCNA. GO analysis of these genes revealed that the function of EZH2/NSD2 axis was associated with cell division, mitotic nuclear division and transition of mitotic cell cycle. These results suggested that EZH2/NSD2 axis may participate in the tumorigenesis and development of TNBC by impacting the process of cell mitosis. KEGG pathway analysis indicated that EZH2/NSD2 axis was connected to the cell cycle pathway. CCNA2-CDK2 complex acts as a core component of cell cycle and often aberrantly expressed in cancer (33). PCNA plays a crucial role in DNA replication during cell cycle progression (34). KDM2B promotes TNBC cell proliferation by repressing the transcription of the cell cycle inhibitors p15INK4B, p16INK4A, and p57KIP2 (35). KIF11 is an essential molecular motor protein for chromosome segregation and mitotic spindle formation. Knockdown of KIF11 causes G2/M phase arrest and inhibits self-renewal in chemotherapy resistant TNBC stem cells (36). KIF23 is responsible for cytoplasm separation and axon elongation, and its high level is significantly correlated with unfavorable overall survival in BC (37). These genes, as essential regulators of the cell division cycle, are usually taken as markers of cell proliferation. Furthermore, recent data suggest that they also function in cell migration and invasion. CCNA2 participates in the modulation of RhoA activity and its dysregulation by autophagy in the late phase of mitosis appears to be associated with EMT, affecting the aggressiveness of the tumor (38). CCNA2 also facilitates EMT *via* the integrin αvβ3 signaling, representing a crucial regulator of tumor cells metastasis (39). Inhibition of CDK2 reduces invasion of prostate cancer cells and reintroduction of CDK2 rescues the invasion ability, indicating that CDK2 is a crucial factor in metastasis of cancer (40). Phosphorylation of SIRT2 at S331 site by CDK2 impairs the catalytic activity of SIRT2, influences microtubule dynamics and antagonizes SIRT2-mediated inhibition of cell motility, including adhesion and migration (41). Positive expression rate of PCNA mRNA in colorectal cancer with liver metastasis was higher than those without liver metastasis, so it may be useful for evaluating liver metastasis of cancer cells (42). KDM2B regulates actin cytoskeleton and epithelial-to-mesenchymal transition by upregulating Rho-GTPases and activating FAK/PI3K signaling, thus promoting tumor cell motility (43–45). KIF11 is a key downstream molecule that responds to directional cues in chemotaxis to govern the direction of migration (46). KIF11 knockdown inhibits the migration and invasion of BC cells *via* decreasing the phosphorylation of ERK, AMPK, AKT, and CREB (47). KIF23 promotes gastric cancer cell proliferation, migration, and invasion by activating Wnt/β-catenin signaling pathway through direct interaction with Amer1 (48). The expression levels of CCNA2, CDK2, and PCNA were reduced in cells with knockdown of EZH2 or NSD2, while the expression levels of KIF11and KIF23 were only reduced in cells with knockdown of EZH2, suggesting that CCNA2, CDK2, and PCNA may be regulated by both EZH2 and NSD2. The proposed mechanism of cell proliferation, migration and invasion regulated by EZH2/NSD2 axis was summarized in **Figure 10**. Our study provided a foundation for investigating the interaction between these six genes and EZH2/NSD2 axis and the mechanism of TNBC growth and metastasis in follow-up work.

Given the important role of EZH2 in tumor pathogenesis, developing small molecule inhibitors of EZH2 becomes popular and clinical trials are underway to test these molecules. Nevertheless, there are still some challenges in direct targeting of EZH2 in cancers due to its various roles. For example, EZH2 plays a major part in normal hematopoietic stem cell differentiation and self-renewal, B cell differentiation, thymopoiesis, and lymphopoiesis (49, 50). Additionally, inactivating mutations or deletion of EZH2 have been identified in several types of tumor, such as Kras-driven lung adenocarcinoma and T cell precursor acute lymphoblastic leukemia, suggesting the tumor-suppressive effect of EZH2 in certain contexts (51, 52). Thus, identifying alternative downstream targets of EZH2 such as NSD2, may provide a new therapeutic strategy.

In summary, the expression of EZH2 and NSD2 are coordinately increased in BC, particularly in TNBC. These two histone methyltransferases act as prognostic indicators for BC patients. EZH2 promotes the proliferation, migration and invasion abilities of TNBC cells *via* upregulating NSD2 expression. EZH2/NSD2 axis may contribute to the progression of TNBC by affecting the cell cycle pathway. Our findings unveiled the role of EZH2/NSD2 histone methyltransferases axis in pathogenesis and progression of TNBC, which may facilitate the development of diagnosis and therapy against TNBC.

## Data Availability Statement

The original contributions presented in the study are included in the article/**Supplementary Material**. Further inquiries can be directed to the corresponding author.

## Ethics Statement

The studies involving human participants were reviewed and approved by the Research Ethics Committee of First Affiliated Hospital of Dali University. The patients/participants provided their written informed consent to participate in this study.

## Author Contributions

BG and YP designed the study. BG, XL, ZL, and LZ acquired interpreted the data. BG and YP drafted and revised the manuscript. All authors contributed to the article and approved the submitted version.

## Funding

This study was supported by the National Natural Science Foundation of China (No. 81960042 and No. 81660037), the Applied Basic Research Project in Yunnan Local Colleges (No. 2018FH001-086), the Applied Basic Research Foundation of Yunnan Province (No. 2016FD072), the Key Laboratory Program in Universities of Yunnan Province (No. 2016037), and the Innovation Team Program of Dali University (No. 2019013).

## Conflict of Interest

The authors declare that the research was conducted in the absence of any commercial or financial relationships that could be construed as a potential conflict of interest.

## Supplementary Material

Supplementary Figure 1 Stable knockdown of EZH2 inhibited the proliferation, migration and invasion of MDA−MB−468 cells, whereas overexpression of EZH2 displayed an inverse phenotype. Stable knockdown or transient overexpression of EZH2 was performed in MDA-MB-468 cells. **(A)** The relative mRNA levels of EZH2 and NSD2 were determined by qRT-PCR. **(B)** The protein levels of EZH2, NSD2 and histone methylation markers were determined by Western blot. Left panel showed the representative images of protein expression and right panel showed the fold changes of protein levels. **(C)** The cell proliferation ability was determined by CCK8 assay. **(D)** The cell migration ability was determined by Wound healing assay. Left panel showed the representative images of cell migration and right panel showed the wound healing rate at 24 h and 48 h. **(E)** The cell invasion ability was determined by Transwell assay. Left panel showed the representative images of cell invasion and right panel showed the absorbance of invasive cells stained by crystal violet. **P* < 0.05, ***P* < 0.01, ****P* < 0.001.

Supplementary Figure 2 Stable knockdown of NSD2 inhibited the proliferation, migration and invasion of MDA−MB−231 and MDA-MB-468 cells. Stable knockdown of NSD2 was performed in MDA-MB-231 and MDA-MB-468 cells. **(A)** The protein levels of EZH2, NSD2 and histone methylation markers were determined by Western blot. Left panel showed the representative images of protein expression and right panel showed the fold changes of protein levels. **(B)** The cell proliferation ability was determined by CCK8 assay. **(C)** The cell migration ability was determined by Wound healing assay. Left panel showed the representative images of cell migration and right panel showed the wound healing rate at 48 h. **(D)** The cell invasion ability was determined by Transwell assay. Left panel showed the representative images of cell invasion and right panel showed the absorbance of invasive cells stained by crystal violet. **P* < 0.05.

Supplementary Figure 3 Knockdown of NSD2 attenuated the proliferation, migration and invasion abilities of EZH2-overexpressing MDA-MB-468 cells. Transient overexpression of EZH2 was performed in MDA-MB-468 cells with stable knockdown of NSD2. **(A)** The relative mRNA levels of EZH2 and NSD2 were determined by qRT-PCR. **(B)** The protein levels of EZH2, NSD2 and histone methylation markers were determined by Western blot. Left panel showed the representative images of protein expression and right panel showed the fold changes of protein levels. **(C)** The cell proliferation ability was determined by CCK8 assay. **(D)** The cell migration ability was determined by Wound healing assay. Left panel showed the representative images of cell migration and right panel showed the wound healing rate at 24 h and 48 h. **(E)** The cell invasion ability was determined by Transwell assay. Left panel showed the representative images of cell invasion and right panel showed the absorbance of invasive cells stained by crystal violet. **P* < 0.05, ***P* < 0.01, NS, non-significant.

## References

[1] Fan L Strasser-Weipp K Li JJ St Louis J Finkelstein DM Yu KD. Breast cancer in China. Lancet Oncol (2014) 15:e279–89. 10.1016/S1470-2045(13)70567-9 24872111

[2] Omarini C Guaitoli G Pipitone S Moscetti L Cortesi L Cascinu S. Neoadjuvant treatments in triple-negative breast cancer patients: where we are now and where we are going. Cancer Manag Res (2018) 10:91–103. 10.2147/CMAR.S146658 29391830 PMC5772398

[3] Tremblay-LeMay R Rastgoo N Pourabdollah M Chang H. EZH2 as a therapeutic target for multiple myeloma and other haematological malignancies. Biomark Res (2018) 6:34. 10.1186/s40364-018-0148-5 30555699 PMC6286605

[4] Gan L Yang Y Li Q Feng Y Liu T Guo W. Epigenetic regulation of cancer progression by EZH2: from biological insights to therapeutic potential. Biomark Res (2018) 6:10. 10.1186/s40364-018-0122-2 29556394 PMC5845366

[5] Yan KS Lin CY Liao TW Peng CM Lee SC Liu YJ. EZH2 in Cancer Progression and Potential Application in Cancer Therapy: A Friend or Foe? Int J Mol Sci (2017) 18:1172. 10.3390/ijms18061172 PMC5485996 28561778

[6] Nakagawa M Kitabayashi I. Oncogenic roles of enhancer of zeste homolog 1/2 in hematological malignancies. Cancer Sci (2018) 109:2342–8. 10.1111/cas.13655 PMC6113435 29845708

[7] Italiano A. Role of the EZH2 histone methyltransferase as a therapeutic target in cancer. Pharmacol Ther (2016) 165:26–31. 10.1016/j.pharmthera.2016.05.003 27179746

[8] Xie Z Chng WJ. MMSET: role and therapeutic opportunities in multiple myeloma. BioMed Res Int (2014) 2014:636514. 10.1155/2014/636514 25093175 PMC4100374

[9] Kuo AJ Cheung P Chen K Zee BM Kioi M Lauring J. NSD2 links dimethylation of histone H3 at lysine 36 to oncogenic programming. Mol Cell (2011) 44:609–20. 10.1016/j.molcel.2011.08.042 PMC3222870 22099308

[10] Keats JJ Maxwell CA Taylor BJ Hendzel MJ Chesi M Bergsagel PL. Overexpression of transcripts originating from the MMSET locus characterizes all t(4;14)(p16;q32)-positive multiple myeloma patients. Blood (2005) 105:4060–9. 10.1182/blood-2004-09-3704 PMC1895072 15677557

[11] García-Carpizo V Sarmentero J Han B Graña O Ruiz-Llorente S Pisano DG. NSD2 contributes to oncogenic RAS-driven transcription in lung cancer cells through long-range epigenetic activation. Sci Rep (2016) 6:32952. 10.1038/srep32952 27604143 PMC5015087

[12] Chen LY Zhi Z Wang L Zhao YY Deng M Liu YH. NSD2 circular RNA promotes metastasis of colorectal cancer by targeting miR-199b-5p-mediated DDR1 and JAG1 signalling. J Pathol (2019) 248:103–15. 10.1002/path.5238 30666650

[13] Han X Piao L Xu X Luo F Liu Z He X. NSD2 promotes renal cancer progression through stimulating Akt/Erk signaling. Cancer Manag Res (2020) 12:375–83. 10.2147/CMAR.S222673 PMC6974414 32021450

[14] Wu J Luo M Duan Z Jia Y Linghu H Tian P. WHSC1 acts as a prognostic indicator and functions as an oncogene in cervical cancer. Onco Targets Ther (2019) 12:4683–90. 10.2147/OTT.S204701 PMC6588087 31354300

[15] Aytes A Giacobbe A Mitrofanova A Ruggero K Cyrta J Arriaga J. NSD2 is a conserved driver of metastatic prostate cancer progression. Nat Commun (2018) 9:5201. 10.1038/s41467-018-07511-4 30518758 PMC6281610

[16] Dai J Jiang L Qiu L Shao Y Shi P Li J. WHSC1 promotes cell proliferation, migration, and invasion in hepatocellular carcinoma by activating mTORC1 signaling. Onco Targets Ther (2020) 13:7033–44. 10.2147/OTT.S248570 PMC7398890 32801739

[17] He C Liu C Wang L Sun Y Jiang Y Hao Y. Histone methyltransferase NSD2 regulates apoptosis and chemosensitivity in osteosarcoma. Cell Death Dis (2019) 10:65. 10.1038/s41419-019-1347-1 30683853 PMC6347630

[18] Asangani IA Ateeq B Cao Q Dodson L Pandhi M Kunju LP. Characterization of the EZH2-MMSET histone methyltransferase regulatory axis in cancer. Mol Cell (2013) 49:80–93. 10.1016/j.molcel.2012.10.008 23159737 PMC3547524

[19] Nohle DG Hackman BA Ayers LW. The tissue micro-array data exchange specification: a web based experience browsing imported data. BMC Med Inform Decis Mak (2005) 5:25. 10.1186/1472-6947-5-25 16086837 PMC1208890

[20] Xu Y He Y Xu W Lu T Liang W Jin W. Promotive effects of capillary morphogenetic protein 2 on glioma cell invasion and the molecular mechanism. Folia Neuropathol (2019) 57:6–15. 10.5114/fn.2019.83826 31038183

[21] Tiffen JC Gunatilake D Gallagher SJ Gowrishankar K Heinemann A Cullinane C. Targeting activating mutations of EZH2 leads to potent cell growth inhibition in human melanoma by derepression of tumor suppressor genes. Oncotarget (2015) 6:27023–36. 10.18632/oncotarget.4809 PMC4694971 26304929

[22] Lauring J Abukhdeir AM Konishi H Garay JP Gustin JP Wang Q. The multiple myeloma associated MMSET gene contributes to cellular adhesion, clonogenic growth, and tumorigenicity. Blood (2008) 111:856–64. 10.1182/blood-2007-05-088674 PMC2200833 17942756

[23] Karsli-Ceppioglu S Dagdemir A Judes G Ngollo M Penault-Llorca F Pajon A. Epigenetic mechanisms of breast cancer: an update of the current knowledge. Epigenomics (2014) 6:651–64. 10.2217/epi.14.59 25531258

[24] Ding L Erdmann C Chinnaiyan AM Merajver SD Kleer CG. Identification of EZH2 as a molecular marker for a precancerous state in morphologically normal breast tissues. Cancer Res (2006) 66:4095–9. 10.1158/0008-5472.CAN-05-4300 16618729

[25] Gong Y Huo L Liu P Sneige N Sun X Ueno NT. Polycomb group protein EZH2 is frequently expressed in inflammatory breast cancer and is predictive of worse clinical outcome. Cancer (2011) 117:5476–84. 10.1002/cncr.26179 21713757

[26] Alford SH Toy K Merajver SD Kleer CG. Increased risk for distant metastasis in patients with familial early-stage breast cancer and high EZH2 expression. Breast Cancer Res Treat (2012) 132:429–37. 10.1007/s10549-011-1591-2 PMC3311223 21614565

[27] Zhao X Xie T Zhao W Cai W Su X. Downregulation of MMSET impairs breast cancer proliferation and metastasis through inhibiting Wnt/beta-catenin signaling. Onco Targets Ther (2019) 12:1965–77. 10.2147/OTT.S196430 PMC6421877 30936716

[28] Wang J Duan Z Nugent Z Zou JX Borowsky AD Zhang Y. Reprogramming metabolism by histone methyltransferase NSD2 drives endocrine resistance via coordinated activation of pentose phosphate pathway enzymes. Cancer Lett (2016) 378:69–79. 10.1016/j.canlet.2016.05.004 27164560 PMC7505026

[29] Kleer CG Cao Q Varambally S Shen R Ota I Tomlins SA. EZH2 is a marker of aggressive breast cancer and promotes neoplastic transformation of breast epithelial cells. Proc Natl Acad Sci U S A (2003) 100:11606–11. 10.1073/pnas.1933744100 PMC208805 14500907

[30] Li X Gonzalez ME Toy K Filzen T Merajver SD Kleer CG. Targeted overexpression of EZH2 in the mammary gland disrupts ductal morphogenesis and causes epithelial hyperplasia. Am J Pathol (2009) 175:1246–54. 10.2353/ajpath.2009.090042 PMC2731143 19661437

[31] Wang JJ Zou JX Wang H Duan ZJ Wang HB Chen P. Histone methyltransferase NSD2 mediates the survival and invasion of triple-negative breast cancer cells via stimulating ADAM9-EGFR-AKT signaling. Acta Pharmacol Sin (2019) 40:1067–75. 10.1038/s41401-018-0199-z PMC6786427 30670815

[32] Yoo KH Hennighausen L. EZH2 methyltransferase and H3K27 methylation in breast cancer. Int J Biol Sci (2012) 8:59–65. 10.7150/ijbs.8.59 22211105 PMC3226033

[33] Gopinathan L Tan SL Padmakumar VC Coppola V Tessarollo L Kaldis P. Loss of Cdk2 and cyclin A2 impairs cell proliferation and tumorigenesis. Cancer Res (2014) 74:3870–9. 10.1158/0008-5472.CAN-13-3440 PMC4102624 24802190

[34] Boehm EM Gildenberg MS Washington MT. The many roles of PCNA in eukaryotic DNA replication. Enzymes (2016) 39:231–54. 10.1016/bs.enz.2016.03.003 PMC4890617 27241932

[35] Zheng Q Fan H Meng Z Yuan L Liu C Peng Y. Histone demethylase KDM2B promotes triple negative breast cancer proliferation by suppressing p15INK4B, p16INK4A, and p57KIP2 transcription. Acta Biochim Biophys Sin (Shanghai) (2018) 50:897–904. 10.1093/abbs/gmy084 30060056

[36] Jiang M Zhuang H Xia R Gan L Wu Y Ma J. KIF11 is required for proliferation and self-renewal of docetaxel resistant triple negative breast cancer cells. Oncotarget (2017) 8:92106–18. 10.18632/oncotarget.20785 PMC5696167 29190901

[37] Song X Zhang T Wang X Liao X Han C Yang C. Distinct diagnostic and prognostic values of kinesin family member genes expression in patients with breast cancer. Med Sci Monit (2018) 24:9442–64. 10.12659/MSM.913401 PMC6322372 30593585

[38] Loukil A Cheung CT Bendris N Lemmers B Peter M Blanchard JM. Cyclin A2: At the crossroads of cell cycle and cell invasion. World J Biol Chem (2015) 6:346–50. 10.4331/wjbc.v6.i4.346 PMC4657123 26629317

[39] Ruan JS Zhou H Yang L Wang L Jiang ZS Wang SM. CCNA2 facilitates epithelial-to-mesenchymal transition via the integrin αvβ3 signaling in NSCLC. Int J Clin Exp Pathol (2017) 10:8324–33. PMC6965382 31966683

[40] Yin X Yu J Zhou Y Wang C Jiao Z Qian Z. Identification of CDK2 as a novel target in treatment of prostate cancer. Future Oncol (2018) 14:709–18. 10.2217/fon-2017-0561 29323532

[41] Pandithage R Lilischkis R Harting K Wolf A Jedamzik B Lüscher-Firzlaff J. The regulation of SIRT2 function by cyclin-dependent kinases affects cell motility. J Cell Biol (2008) 180:915–29. 10.1083/jcb.200707126 PMC2265402 18332217

[42] Yue S-Q Yang Y-L Dou K-F Li K-Z. Expression of PCNA and CD44mRNA in colorectal cancer with venous invasion and its relationship to liver metastasis. World J Gastroenterol (2003) 9:2863–5. 10.3748/wjg.v9.i12.2863 PMC4612073 14669354

[43] Zacharopoulou N Tsapara A Kallergi G Schmid E Tsichlis PN Kampranis SC. The epigenetic factor KDM2B regulates cell adhesion, small rho GTPases, actin cytoskeleton and migration in prostate cancer cells. Biochim Biophys Acta Mol Cell Res (2018) 1865:587–97. 10.1016/j.bbamcr.2018.01.009 29408056

[44] Zacharopoulou N Tsapara A Kallergi G Schmid E Alkahtani S Alarifi S. The epigenetic factor KDM2B regulates EMT and small GTPases in colon tumor cells. Cell Physiol Biochem (2018) 47:368–77. 10.1159/000489917 29772566

[45] Zacharopoulou N Kallergi G Alkahtani S Tsapara A Alarifi S Schmid E. The histone demethylase KDM2B activates FAK and PI3K that control tumor cell motility. Cancer Biol Ther (2020) 21:533–40. 10.1080/15384047.2020.1736481 PMC7515453 32175798

[46] Wang F Lin SL. Knockdown of kinesin KIF11 abrogates directed migration in response to epidermal growth factor-mediated chemotaxis. Biochem Biophys Res Commun (2014) 452:642–8. 10.1016/j.bbrc.2014.08.136 25193695

[47] Zhou J Chen W-R Yang L-C Wang J Sun J-Y Zhang W-W. KIF11 functions as an oncogene and is associated with poor outcomes from breast cancer. Cancer Res Treat (2019) 51:1207–21. 10.4143/crt.2018.460 PMC6639218 30590004

[48] Liu Y Chen H Dong P Xie G Zhou Y Ma Y. KIF23 activated Wnt/β-catenin signaling pathway through direct interaction with Amer1 in gastric cancer. Aging (Albany NY) (2020) 12:8372–96. 10.18632/aging.103146 PMC7244035 32365332

[49] Good-Jacobson KL. Regulation of germinal center, B-cell memory, and plasma cell formation by histone modifiers. Front Immunol (2014) 5:596. 10.3389/fimmu.2014.00596 25477884 PMC4237133

[50] Karantanos T Chistofides A Barhdan K Li L Boussiotis VA. Regulation of T Cell Differentiation and Function by EZH2. Front Immunol (2016) 7:172. 10.3389/fimmu.2016.00172 27199994 PMC4853381

[51] Zhang J Ding L Holmfeldt L Wu G Heatley SL Payne-Turner D. The genetic basis of early T-cell precursor acute lymphoblastic leukaemia. Nature (2012) 481:157–63. 10.1038/nature10725 PMC3267575 22237106

[52] Wang Y Hou N Cheng X Zhang J Tan X Zhang C. Ezh2 Acts as a Tumor Suppressor in Kras-driven Lung Adenocarcinoma. Int J Biol Sci (2017) 13:652–9. 10.7150/ijbs.19108 PMC5441181 28539837

