# Supplementary figures and images for: Overexpression of EZH2/NSD2 Histone Methyltransferase Axis Predicts Poor Prognosis and Accelerates Tumor Progression in Triple-Negative Breast Cancer
Source: Front Oncol. 2021 Feb 16;10:600514. doi: 10.3389/fonc.2020.600514 (PMC7921704; doi:10.3389/fonc.2020.600514)

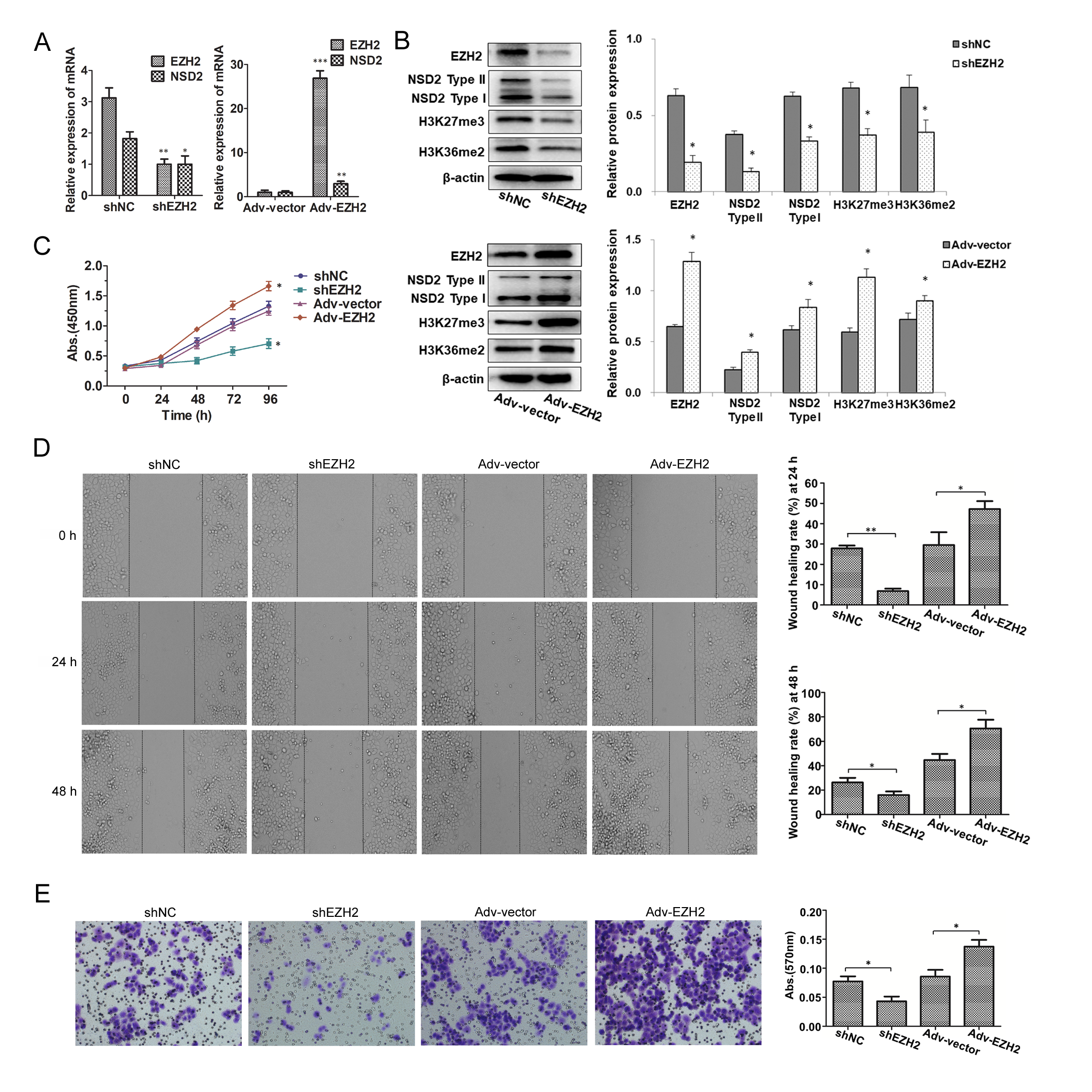

Supplement: Supplementary Figure 1 — Stable knockdown of EZH2 inhibited the proliferation, migration and invasion of MDA−MB−468 cells, whereas overexpression of EZH2 displayed an inverse phenotype. Stable knockdown or transient overexpression of EZH2 was performed in MDA-MB-468 cells. (A) The relative mRNA levels of EZH2 and NSD2 were determined by qRT-PCR. (B) The protein levels of EZH2, NSD2 and histone methylation markers were determined by Western blot. Left panel showed the representative images of protein expression and right panel showed the fold changes of protein levels. (C) The cell proliferation ability was determined by CCK8 assay. (D) The cell migration ability was determined by Wound healing assay. Left panel showed the representative images of cell migration and right panel showed the wound healing rate at 24 h and 48 h. (E) The cell invasion ability was determined by Transwell assay. Left panel showed the representative images of cell invasion and right panel showed the absorbance of invasive cells stained by crystal violet. *P < 0.05, **P < 0.01, ***P < 0.001. [file Image_1.tif]

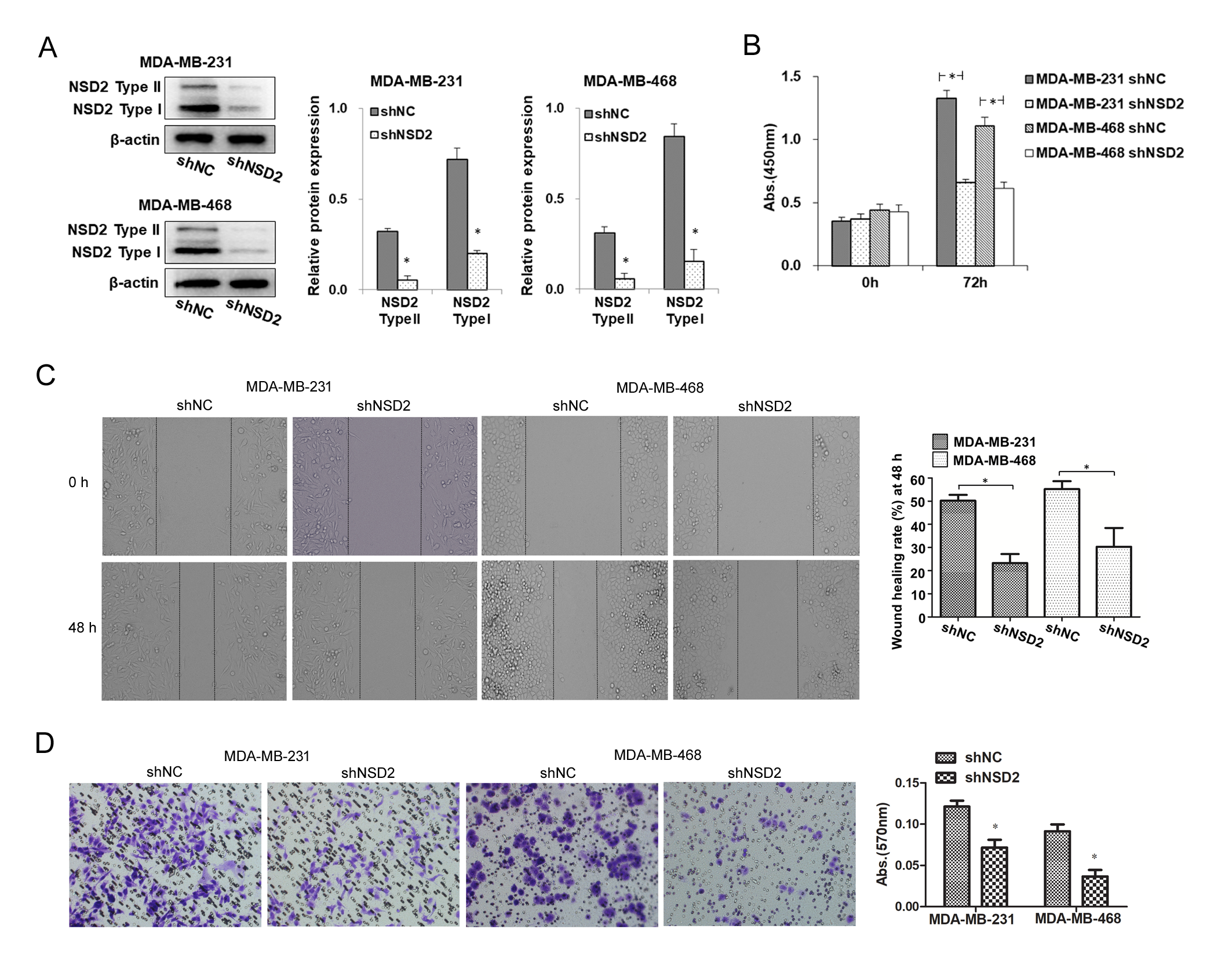

Supplement: Supplementary Figure 2 — Stable knockdown of NSD2 inhibited the proliferation, migration and invasion of MDA−MB−231 and MDA-MB-468 cells. Stable knockdown of NSD2 was performed in MDA-MB-231 and MDA-MB-468 cells. (A) The protein levels of EZH2, NSD2 and histone methylation markers were determined by Western blot. Left panel showed the representative images of protein expression and right panel showed the fold changes of protein levels. (B) The cell proliferation ability was determined by CCK8 assay. (C) The cell migration ability was determined by Wound healing assay. Left panel showed the representative images of cell migration and right panel showed the wound healing rate at 48 h. (D) The cell invasion ability was determined by Transwell assay. Left panel showed the representative images of cell invasion and right panel showed the absorbance of invasive cells stained by crystal violet. *P < 0.05. [file Image_2.tif]

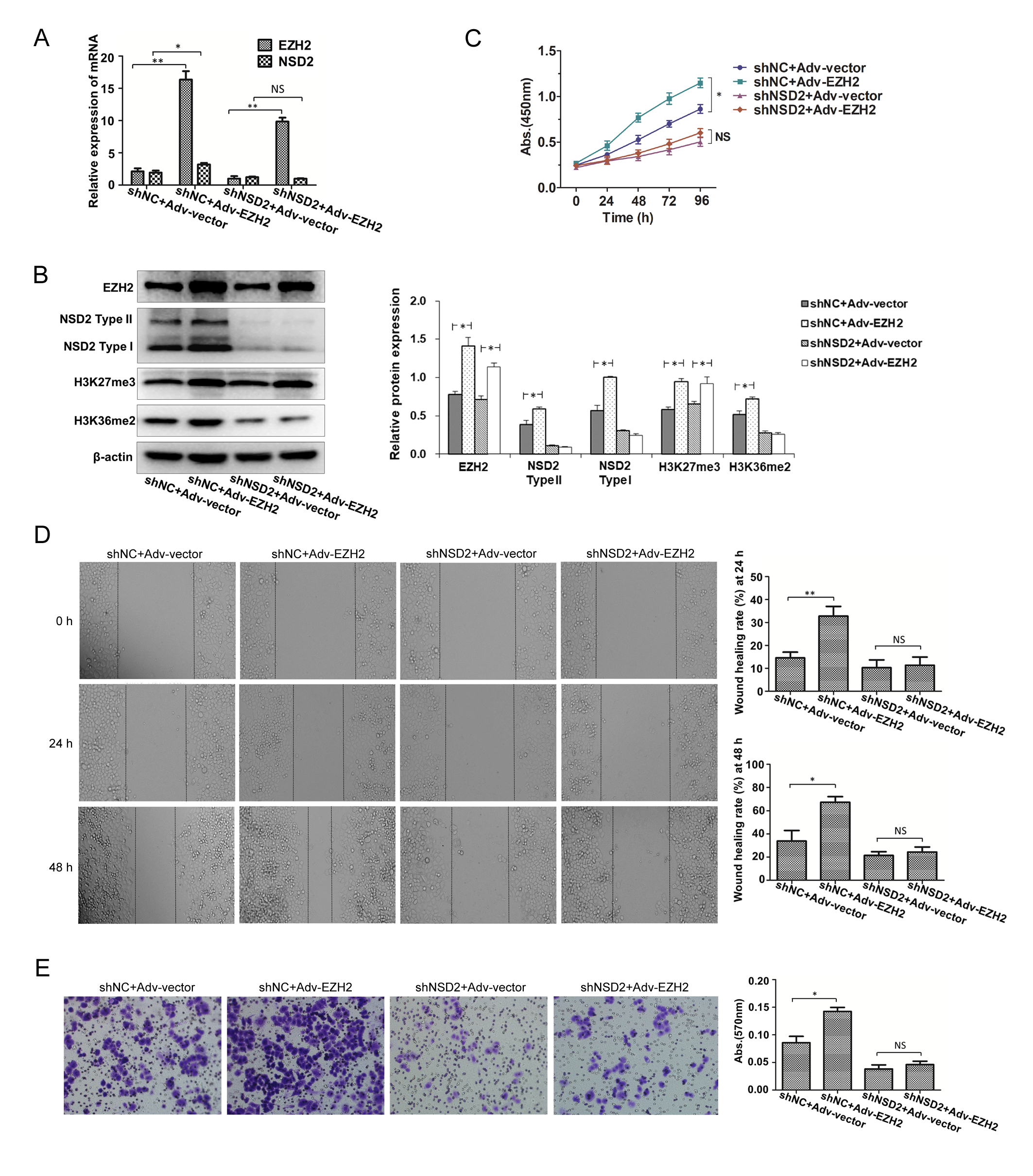

Supplement: Supplementary Figure 3 — Knockdown of NSD2 attenuated the proliferation, migration and invasion abilities of EZH2-overexpressing MDA-MB-468 cells. Transient overexpression of EZH2 was performed in MDA-MB-468 cells with stable knockdown of NSD2. (A) The relative mRNA levels of EZH2 and NSD2 were determined by qRT-PCR. (B) The protein levels of EZH2, NSD2 and histone methylation markers were determined by Western blot. Left panel showed the representative images of protein expression and right panel showed the fold changes of protein levels. (C) The cell proliferation ability was determined by CCK8 assay. (D) The cell migration ability was determined by Wound healing assay. Left panel showed the representative images of cell migration and right panel showed the wound healing rate at 24 h and 48 h. (E) The cell invasion ability was determined by Transwell assay. Left panel showed the representative images of cell invasion and right panel showed the absorbance of invasive cells stained by crystal violet. *P < 0.05, **P < 0.01, NS, non-significant. [file Image_3.tif]
